# Problems and Approaches in the Improvement of Gluten‐Free Bread Texture: A Comprehensive Review

**DOI:** 10.1155/ijfo/5214023

**Published:** 2026-05-11

**Authors:** Zaripa Alibekova, Meruert Bayisbayeva, Rosnah Shamsudin, Asem Bakhtybekova, Ravshanbek Alibekov, Zhambul Aimenov

**Affiliations:** ^1^ Food Chemistry and Biotechnology Scientific-Research Laboratory, M. Auezov South Kazakhstan University, Shymkent, Kazakhstan; ^2^ Technology of Bread Products and Processing Industries Department, Almaty Technological University, Almaty, Kazakhstan; ^3^ Department of Process and Food Engineering, Faculty of Engineering, Universiti Putra Malaysia, Serdang, Selangor, Malaysia, upm.edu.my

**Keywords:** dough, enzymes, hydrocolloids, process, proteins, quality, structure

## Abstract

Currently, creating gluten‐free bread comparable with traditional bread remains a major scientific and technological challenge. Gluten is a wheat flour protein complex, mainly gliadins (*ω*‐, *α*‐, and *γ*‐gliadins) and glutenins consist of low molecular–weight and high molecular–weight subunits. The absence of a gluten framework negatively impacts the dough structure and texture. This comprehensive analysis is aimed at identifying the most effective and industrially relevant approaches for structuring gluten‐free bread texture and practical implementation. Valuable attention is given to combinations of alternative protein flours (rice, sorghum, legumes, and pseudocereals) used to enrich gluten‐free products with plant hydrocolloids, including pectin, *β*‐glucan, gum arabic, gum tragacanth, guar gum, psyllium, and locust bean gum; and animal‐derived hydrocolloids: gelatin and milk proteins (caseinate and whey concentrates). The following main review directions are discussed: hydrocolloids structuring and moisture retention, protein ingredients and protein–hydrocolloid interactions, fermentation and enzyme preparations, staling of gluten‐free bread and methods of slowing it down, extrusion and microencapsulation technologies, and innovations and future directions. The problems of scalability and economic feasibility of the latest approaches; advances in food chemistry, biotechnology, process engineering, and consumer science; prospects for further research in the improvement of gluten‐free bread value and meeting the growing consumer demand for healthy food products are considered.

## 1. Introduction

In recent decades, the number of people who must or choose to avoid gluten has increased markedly that has stimulated the development of high‐quality gluten‐free bakery products [1–3]. Celiac disease is recognized as one of the most common lifelong disorders of the digestive system, with a prevalence of about 1% of the global population [4]. Due to improved diagnostics and greater awareness, the detection of celiac disease is rising, although many cases remain undiagnosed [5, 6]. In addition, nonceliac gluten sensitivity (NCGS) is receiving growing attention, with reported prevalence estimates ranging from roughly 0.5% to 13%. Even individuals without a formal diagnosis increasingly restrict gluten intake for perceived health benefits: In some specific groups, such as athletes and fitness‐oriented consumers, up to 30%–50% of respondents report voluntarily avoiding gluten‐containing products [1, 2, 7]. As a result, the demand for gluten‐free bread is now driven not only by medical indications but also by a broader consumer interest in “gluten‐free” as a symbol of a healthy diet [2].

The food industry is actively responding to this demand. The global gluten‐free products market size was estimated at $7.75 billion in 2024 and is projected to reach $13.67 billion by 2030, growing at a CAGR of 10.0% from 2025 to 2030. The gluten‐free product industry has seen significant growth in recent years, driven by increasing awareness of gluten sensitivities, celiac disease, and the broader health‐conscious consumer base [8]. The European market is also expanding, for instance, the sales of gluten‐free products was around €3.62 billion in 2024 [9]. This expansion is mainly driven by the increasing diagnosis of gluten‐related disorders, greater consumer awareness (e.g., EU Regulation No. 609/2013 on the labelling of gluten‐free foods) and the perception of gluten‐free products as healthier alternatives, even among individuals without a medical diagnosis [10–12]. Against this background, gluten‐free baked goods have become an important market segment, and their technological and sensory quality directly determines the competitiveness of manufacturers.

Despite the growing market, achieving the quality of gluten‐free bread comparable with traditional bread remains a complex scientifical and technological challenge. The absence of the gluten framework formed by gluten has a negative impact on the structure of the dough and the finished product [13, 14]. When kneaded, gluten forms a three‐dimensional elastic network capable of retaining gas and providing volume and an elastic crumb texture. In the absence of a gluten network, gluten‐free bread is usually characterized by a smaller volume and a denser crumb because the dough cannot effectively retain gas and moisture. Surveys show that up to 70% of consumers are dissatisfied with the quality of gluten‐free bread, noting its rough, crumbly structure, and differences in taste [15, 16]. Indeed, the crumb of gluten‐free bread is less elastic, goes stale faster, and crumbles easily, and its organoleptic properties are often inferior to those of its wheat counterpart. To improve taste and shelf life, some manufacturers are forced to add fats and sugars or enhance the recipe with starches, which increases the caloric content and glycemic index of the product [1, 17]. These compromises, along with the high prices of gluten‐free ingredients, reduce the attractiveness of the product, and highlight the urgency of finding new solutions [18–20].

Recently, various approaches to improving the quality and texture of gluten‐free bread have been actively studied. In order to imitate the properties of gluten, hydrocolloids (xanthan and guar gums, carboxymethyl cellulose, psyllium, etc.) are introduced into the recipes [21–23], increasing dough viscosity and improving gas retention during fermentation. The use of hydrocolloids has proven to be effective in increasing crumb volume and porosity; for example, a combination of xanthan and guar gums produces a more uniform bread structure and greater volume than using either alone [16–18]. To strengthen the protein–starch matrix of gluten‐free dough, additional proteins are used—milk (whey), egg, as well as plant isolates: soy, pea, and others [24]. Protein supplements increase nutritional value and assist in the structure form, but excess plant protein can cause off‐flavors and impair the consistency of the product [25]. Enzymatic modification of dough is considered a promising direction: The addition of transglutaminase (TG) to cross‐link proteins increases the elasticity of gluten‐free dough [26], and amylolytic and proteolytic enzymes improve crumb softness and release aromatic compounds [27]. At the same time, the technology of sourdoughs based on lactic acid bacteria is developing: Fermentation promotes the accumulation of natural hydrocolloids (exopolysaccharides) and organic acids that improve the structure and taste of bread [28]. In order to overcome technological barriers, innovative methods of raw material processing are also being explored—extrusion of grain masses that changes the rheological properties of flour [29], ultrasound exposure during kneading, and microencapsulation of functional additives for their uniform distribution [30]. The listed approaches have already made it possible to significantly bring the quality of gluten‐free bread closer to traditional bread. However, none of the methods can completely replace the complex effect of gluten, so in practice, complex recipes with several types of additives are used. Each technology has limitations—from increased product costs to impact on taste—which confirms the need for further research and improvement of the recipe composition.

Thus, the problem of improving the texture of gluten‐free bread remains highly relevant from both scientific and applied points of view. The continuing growth of the gluten‐free market requires manufacturers to develop products that not only meet strict dietary requirements but also meet consumer expectations in terms of softness, elasticity, flavor, and price [31].

The present review is intended to fill a specific gap in the literature by bringing together, within a single framework, both ingredient‐based and process‐based strategies for improving gluten‐free bread texture. In contrast to most earlier reviews, which typically focused either on formulation aspects (e.g., hydrocolloids, proteins, and enzymes) or on individual technologies (e.g., sourdough and extrusion), this work provides an integrated synthesis of how these approaches interact to influence key texture parameters such as specific loaf volume, crumb hardness, elasticity, and staling behavior. Particular attention is paid for the studies published between 2021 and 2026 and to innovative solutions such as extrusion, ultrasound‐assisted processing, and biotechnological starters, which have so far been discussed only in a fragmented way. This comprehensive analysis is aimed at identifying the most effective and industrially relevant strategies for structuring gluten‐free bread and to outline directions for further research and practical implementation.

## 2. Literature Review: Search Strategy and Texture Challenges in Gluten‐Free Bread

### 2.1. Database Analysis

For the review, publications from the Scopus, Web of Science, PubMed, Science Direct, and Google Scholar databases for the period 2021–2026 were analyzed. At the first stage of data collection, the texts of titles and abstracts were analyzed to identify relevant publications. In the subsequent search, all identified keywords and corresponding indicators were reviewed. As a result, articles with the keywords gluten‐free bread, hydrocolloids, fermentation, enzymes, microencapsulation were selected. In addition, the reference lists of all sources accepted for analysis were reviewed, including original and review articles, book chapters, electronic publications, and other scientific materials. The obtained data were assessed and systematized.

### 2.2. The Main Problems of Gluten‐Free Bread Texture

**Figure 1:**
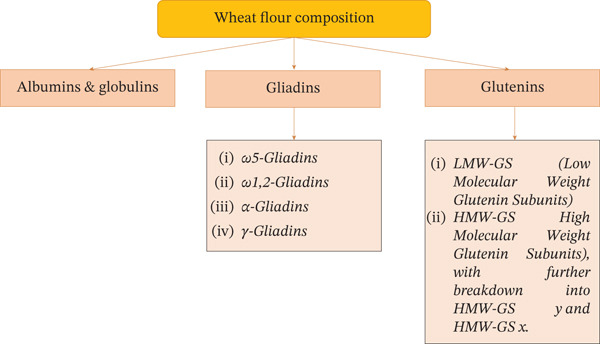
Classification of wheat proteins.

Gluten is a unique protein complex of wheat flour composed mainly of gliadins and glutenins (Figure 1). Gliadins (*ω*‐, *α*‐, *γ*‐gliadins) are predominantly monomeric proteins and contribute to dough extensibility, whereas glutenins consist of polymeric low molecular–weight (LMW) and high molecular–weight (HMW) subunits linked by disulfide bridges that are primarily responsible for dough elasticity and structural stability [32–35].

Classification and molecular weight of wheat gluten proteins are presented in Table 1. The resulting viscoelastic gluten network allows wheat dough to retain gas during fermentation and baking and to form an elastic, cohesive crumb structure.

**Table 1:** Classification and molecular weight of wheat gluten proteins.

Gluten protein type	Forms of the proteins	Group	Molecular weight
*ω*5‐Gliadins	Monomeric	Medium molecular weight	50,900 Da
*ω*1,2‐Gliadins	Monomeric	Medium molecular weight	43,500 Da
*α*‐Gliadins	Monomeric	Low molecular weight	31,800 Da
*γ*‐Gliadins	Monomeric	Low molecular weight	35,200 Da
LMW‐GS	Polymeric	Low molecular weight	32,000 Da
HMW‐GS *y*	Polymeric	High molecular weight	68,700 Da
HMW‐GS *x*	Polymeric	High molecular weight	86,800 Da

In gluten‐free doughs, this protein matrix is absent or strongly reduced, which leads to a pronounced loss of cohesion and elasticity [36]. As a consequence, gluten‐free breads often show lower specific loaf volume, denser and crumbly crumb structure, and a reduced capacity to retain gas, especially during proofing and early baking [37]. Such texture does not match consumer expectations formed by conventional wheat bread that is characterized by a soft, elastic, and “airy” crumb. Firmness (hardness), cohesiveness, and springiness (elasticity) are the main representative parameters of the bread sample texture. Texture profile analysis (TPA) is generally performed by using a texture analyzer device.

**Figure 2:**
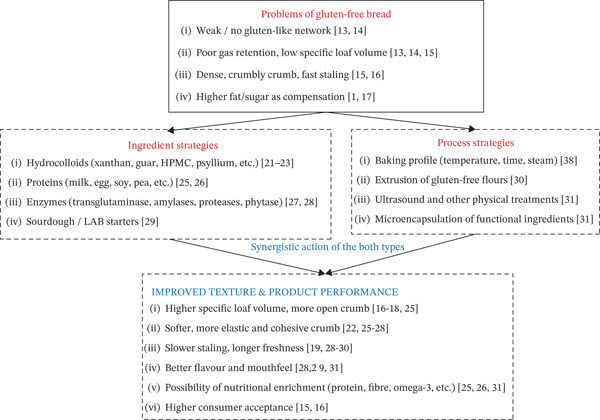
Conceptual overview of problems and approaches in gluten‐free bread texture.

These relationships between the typical problems of gluten‐free dough, the ingredient‐ and process‐based approaches used to address them, and the resulting improvements in bread texture are schematically summarized in Figure 2.

From a technological point of view, the key challenge is therefore to reconstruct, at least partially, the mechanical strength and gas‐retaining capacity of the dough without gluten. This is usually attempted by combining different groups of structure‐forming agents such as starch‐rich flours, protein ingredients, and hydrocolloids, often together with suitable emulsifiers and leavening agents. The main raw materials used for this purpose in gluten‐free flour blends are summarized in Table 2.

**Table 2:** Main raw materials used in gluten‐free flour mixes.

No.	Main groups of structure‐forming agents	Raw materials included in certain groups of structure‐forming agents	Related references
I	Flour with a high content of starch and nonstarch polysaccharides	Rice flour, corn flour, oat flour, pseudocereal flour (amaranth and buckwheat) and cereal flour (millet), sorghum flour, flaxseed flour, peanut flour, lupine flour, etc.	[20, 24, 38, 39]
II	High protein ingredients	Soy isolates and concentrates, pea protein isolates, lupine, caseinates, whey protein concentrates, etc.	[23, 40–42]
III	Hydrocolloids	Xanthan, guar gum, various types of natural and modified starches (potato, corn, rice, sorghum, etc.), and microbial polysaccharides.	[19, 43–45]
IV	Emulsifiers, leavening agents, flavoring ingredients	Melange, lecithin, baking soda, salt, sugar, flavors, colors, and mineral additives.	[37, 46–48]

In addition to ingredient selection, processing conditions and technological treatments also play an important role. Thermal modification and extrusion, as well as the use of specific enzyme preparations, can modify starch and nonstarch polysaccharides, improve water distribution in the dough, and enhance gas retention. These aspects, including the functional roles of hydrocolloids, proteins, and enzymes, are discussed in more detail in Section 3.

## 3. Integrated Approaches to Structuring and Extending Freshness of Gluten‐Free Bread

Gluten‐free bread is noticeably different from traditional wheat bread due to the absence of the gluten framework responsible for the elasticity and flexibility of the dough. This requires finding solutions that will ensure optimal volume, softness, and preservation of freshness of the product [44].

Key areas for texture improvement include as follows:

• Application of hydrocolloids.
• Introduction of proteins.
• Using enzymes, starters and fermentation.
• Sensory acceptability and a staling control.
• latest technologies′ application.

### 3.1. Hydrocolloids: Structuring and Moisture Retention

Normally, wheat bread dough is viscoelastic or shapeable. However, gluten‐free bread dough should be shapeable and sometimes semiliquid, pourable, or batter consistency.

**Figure 3:**
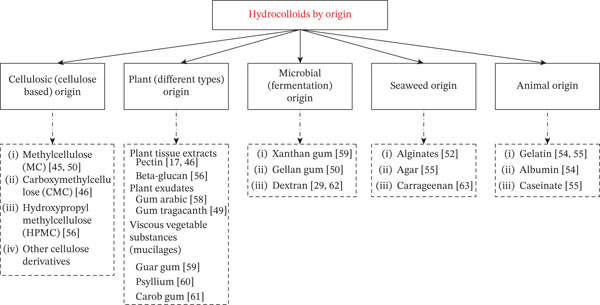
Hydrocolloids by origin.

Hydrocolloids are central to the structuring of gluten‐free doughs because they partly replace the viscoelastic function of gluten. By increasing batter viscosity and water‐holding capacity, they stabilize gas bubbles during proofing and baking and reduce the rate of moisture migration during storage. As a consequence, appropriately selected hydrocolloid systems can increase specific volume, improve crumb softness and resilience, and delay staling [17, 44]. In this review, hydrocolloids are classified according to their origin (Figure 3), since this classification broadly reflects their molecular structure and technological role in gluten‐free bread.

Cellulose derivatives such as methylcellulose, carboxymethylcellulose (CMC), and hydroxypropyl methylcellulose (HPMC) are among the most widely used hydrocolloids in gluten‐free bread. At levels of about 0.5%–2.0%, they markedly increase batter viscosity and gas‐holding capacity, yielding breads with higher specific volume and softer crumb; nevertheless, too high concentrations can produce dense or sticky crumbs [44, 45].

Natural polysaccharides, particularly plant‐derived hydrocolloids, including pectin, *β*‐glucan, gum arabic, gum tragacanth, guar gum, psyllium, and locust bean gum (LBG), provide both technological and nutritional benefits. Besides thickening the dough and reinforcing the cell walls, they contribute soluble dietary fiber and may lower the glycaemic response of gluten‐free bread [49, 50]. However, their sensory impact is complex; for example, high levels of psyllium can lead to a rubbery crumb texture, whereas carob and gum tragacanth may increase stickiness or impart a characteristic flavor.

Microbial hydrocolloids derived from fermentation, such as xanthan and gellan gums as well as exopolysaccharides produced in situ by lactic acid bacteria, are effective even at low dosages. They are particularly attractive for clean‐label formulations because they can be generated during sourdough fermentation and can act synergistically with cereal or plant fibers to stabilize the crumb [14, 49]. Seaweed‐based hydrocolloids (alginates, agar, and carrageenan) have received less attention in gluten‐free bread, but their strong gel‐forming properties make them promising for specific applications, for example in flat breads or products with high moisture [17, 51].

Other protein‐based or animal‐derived hydrocolloids such as gelatin and milk proteins (caseinate and whey concentrates) can form thermally stable gels and protein–polysaccharide complexes that support the crumb structure, although their use is constrained by dietary preferences and labelling issues [52, 53]. Figure 3 summarizes this classification by origin and highlights the main examples relevant to gluten‐free bread. Rather than providing an exhaustive description of each hydrocolloid, the following sections focus on how specific types and combinations influence texture attributes such as specific volume, crumb hardness, elasticity, and crumbliness (see also Table 3 for typical concentration ranges and main effects).

**Table 3:** Types of hydrocolloids and recommended concentrations.

Hydrocolloid	Recommended concentration (%), in total volume	Main effect	Source
CMC (carboxymethylcellulose)	0.5–1.5	Increases viscosity, improves crumb structure	[45, 54, 55]
HPMC (hydroxypropyl methylcellulose)	0.5–1.0 (approximately)	Prolongs freshness, increases gas retention	[44, 45, 54, 56]
Pectin	0.5–2.0	Increases softness and elasticity, reduces GI	[17, 44, 50]
Beta‐glucan	1.0–3.0	Increases volume, retains moisture	[49, 57]
Gum arabic	1.5–3.0	Stabilizes dough, increases softness and shelf life	[48, 53, 54]
Gum tragacanth	0.5–1.5	Prevents delamination, improves gas retention	[48, 50]
Xanthan + guar gum	0.5–1.5 (in total)	Retain gas, increase volume, improve crumb porosity	[37, 54, 56, 58]
Guar gum	0.5–1.0	Increases viscosity, distributes gas bubbles	[54, 58, 59]
Psyllium	1.0–2.5	Improves volume and elasticity, excess gives a “rubber” texture	[56, 60]
Carob gum	0.5–1.5	Makes the dough smoother and more airy	[37, 58, 61]
Broccoli leaf powder	3–6	Increases protein, mineral and antioxidant content	[62]
Artichoke flour	7	Increases the proportion of dietary fiber and polyphenols	[63]

Hydrocolloids by polymeric structures stabilize the structure, improve crumb porosity, and prolong the shelf life of the final gluten free bread. They stabilize the structure, improve crumb porosity, organoleptic properties, and eatability [17]. Cellulose derivatives (MC, CMC, HPMC, and HEC), as well as plant gums (pectin, beta‐glucan, gum arabic, psyllium, etc.) form a more elastic bread structure [44, 52, 64].

Alternative plant ingredients (e.g., broccoli leaf powder, artichoke flour, and okra) at levels of 3%–7% further enhance nutritional value and influence texture through pectin and dietary fiber [46, 55, 65].

The addition of HPMC results in the most significant increase in the elastic/viscosity modulus of the dough compared with other hydrocolloids due to the ability of HPMC to form a foam matrix and to trap gas. Xanthan and guar gums are also widely used; their combination acts synergistically—the combined addition of XG + GG produces a softer crumb (lower crumb hardness) and a higher specific volume of bread than either gum alone [17]. Guar gum, being highly hydrophilic, slows down staling by retaining moisture and inhibiting starch retrogradation. Psyllium (psyllium husk) acts as an effective structure‐forming and antistaling agent: In experiments, it provided the softest and most elastic crumb among all additives and significantly reduced the rate of bread staling [44]. Table 3 provides quantitative data from the literature for illustrating the effect of hydrocolloids and related additives on the quality of gluten‐free bread:

Thus, the use of hydrocolloids has proven effective in improving the quality of gluten‐free bread: They increase loaf volume and crumb porosity by enhancing gas retention and slow down staling through water binding in the crumb structure [17, 20, 64]. For example, adding about 0.5%–1.5% xanthan + guar gum can increase the specific loaf volume by approximately 10%–15% and significantly reduce crumb hardness [37, 58]. The addition of 2%–3% HPMC often allows the specific volume and softness of gluten‐free bread to approach those of control wheat bread [43, 54]. Psyllium (around 5% of flour weight) has been reported to increase the specific volume by 2–3 times and markedly slow down bread staling during storage [44]. Importantly, hydrocolloids simultaneously serve as sources of dietary fiber, thereby increasing the nutritional value of gluten‐free baked goods [57].

However, excessive levels of hydrocolloids or inappropriate combinations may cause detrimental effects, such as excessive dough viscosity, rubbery crumb texture, or off‐flavors, which again highlights the need to optimize hydrocolloid type and dosage [6, 66]. In particular, although xanthan and guar gums together usually soften the crumb, literature data indicate that combining HPMC with guar gum at high concentrations can increase bread hardness [43]. Overall, hydrocolloid systems are highly effective when properly dosed and are almost indispensable for improving the structure of gluten‐free dough.

### 3.2. Protein Ingredients and Protein–Hydrocolloid Interactions

Protein ingredients are used in gluten‐free formulations not only to increase nutritional value but also as additional network‐forming agents that can partially compensate for the lack of gluten. From a textural point of view, proteins primarily influence specific loaf volume, crumb hardness, and elasticity by contributing to gas‐cell stabilization, film formation around starch granules, and modulation of dough rheology [62, 63]. Their effect, however, strongly depends on protein source, solubility, and dosage.

Animal proteins, especially milk and egg proteins, have been repeatedly shown to improve crumb structure when used at moderate levels. Enriching gluten‐free dough with dairy products (e.g., fresh cottage cheese or whey protein concentrates) can increase loaf volume and crumb softness, as denatured whey and casein proteins form a supporting network that stabilizes gas bubbles and reinforces the crumb skeleton [39, 53]. For instance, the addition of 5%–20% fresh cottage cheese (based on flour weight) has been reported to increase loaf volume by up to 73% and reduce crumb hardness by about 65% compared with a control formulation, while slowing down staling by approximately 70% [53]. At the microstructural level, casein and whey proteins, as well as egg proteins, form continuous films around starch granules and gas cells, which brings the crumb closer to a mesh‐like structure that is more similar to wheat bread [39, 40].

Egg albumen is a particularly powerful structure‐forming ingredient due to its foaming and film‐forming properties. At levels of about 5%–10% of the dry component weight, egg white protein increases specific loaf volume, improves crumb porosity, and enhances elasticity and shape stability by forming an elastic protein matrix that traps and stabilizes gas bubbles during proofing and baking [40]. Nevertheless, very high additions can lead to a rubbery crumb or excessive crust browning because of intensive protein denaturation and Maillard reactions.

Plant proteins (soy, pea, rice, lupine, hemp, rapeseed, etc.) attract growing attention as functional ingredients that increase protein content and improve amino acid balance in gluten‐free bread [41, 67]. Their impact on texture is more complex and often dose‐dependent. Rapeseed protein isolate (≈96% protein) at about 10% of flour weight has been shown to improve pasting properties, increase loaf volume, and reduce crumb hardness during storage [41]. However, several studies indicate that additions above 10% of soy or pea isolates can decrease loaf volume and increase hardness, presumably because excess unbound proteins compete with starch for water and interfere with starch gelatinization [38, 66]. Lupine protein at similar levels often shows a weaker effect on structure [66]. In addition, plant proteins rich in lysine (e.g., pea and hemp) intensify Maillard browning during baking; some of the resulting aroma compounds may not be positively perceived by consumers [42, 66]. This highlights the need to balance nutritional benefits against potential adverse effects on texture and flavor.

Synergistic effects are frequently observed when proteins are combined with hydrocolloids. Hydrocolloids bind water and increase batter viscosity, whereas proteins form films and networks that stabilize gas cells; together, they create a more continuous and resilient structure than either component alone. For example, the combined use of 5%–10% egg white and xanthan gum has been reported to increase specific loaf volume and reduce crumb hardness and staling more effectively than either additive alone [66]. Collagen (gelatin) can be even more effective than egg albumen in softening the crumb and delaying firming [53]. On the other hand, too high overall protein levels or suboptimal protein‐to‐hydrocolloid ratios can result in dense, hard crumbs, crust darkening, and beany or off‐flavors in the case of some legume proteins [42, 53]. During baking, protein–hydrocolloid mixtures set into an interconnected gel network as proteins denature and hydrocolloids hydrate and gel, which ultimately defines crumb cell size and elasticity. Therefore, optimizing both the level and type of protein, as well as its combination with hydrocolloids, is crucial to obtain gluten‐free bread with higher specific volume, softer crumb, and improved resilience, while preserving desirable color and flavor.

### 3.3. Enzymes, Starters, and Fermentation Using

Starters and enzymes are key biotechnological tools for modifying gluten‐free dough structure. They influence not only gas production and flavor but also crumb texture attributes such as specific volume, hardness, and cell uniformity. Lactic acid fermentation using gluten‐free sourdoughs based on *Lactobacillus* spp.*, Bifidobacterium* spp., and yeast improves dough rheology and sensory quality by producing organic acids, exopolysaccharides, and endogenous enzymes [14]. These metabolites contribute to a finer and more homogeneous crumb structure, better moisture retention, and delayed firming during storage. Gluten‐free sourdoughs also enhance aroma and impart “artisanal” notes, whereas the lowered pH slows microbiological spoilage. Importantly, several studies have shown that textural benefits (e.g., lower crumb hardness and higher elasticity) are most pronounced when sourdough is combined with targeted enzymes rather than applied alone [14, 68].

In addition to lactic acid bacteria, traditional baker′s yeast (*Saccharomyces cerevisiae*) plays a central role in gas production and crumb structure. In gluten‐free doughs, yeast activity is strongly affected by the availability of fermentable sugars and by batter viscosity. Adequate yeast fermentation leads to the formation of numerous gas cells and higher specific loaf volume, but in weak or low‐viscosity systems it may also cause bubble coalescence and collapse if gas cells are not stabilized by hydrocolloids or proteins [14, 69]. This means that yeast performance must be optimized together with structuring agents to obtain a fine, uniform crumb; simply increasing fermentation time or yeast level does not necessarily translate into better texture.

Separately added enzyme preparations assist to compensate for the low endogenous enzyme activity of gluten‐free flours and the weak dough structure. The most commonly used enzyme groups are amylolytic, proteolytic, TG, phytase, and enzymes produced during sourdough fermentation [14]. Amylolytic enzymes (*α*‐amylase and glucoamylase) are usually added in small doses (about 0.01%–0.1%) [68]. They hydrolyze part of the starch into dextrins and simple sugars, thereby improving fermentation conditions (more fermentable sugars for yeast) and softening the crumb by modifying the starch phase and slowing down amylopectin retrogradation [70, 71]. However, excessive amylase levels can lead to gummy or sticky crumbs and reduced loaf volume, underlining the need for precise dosing.

Proteases (typically 0.1%–0.3% of flour weight) partially hydrolyze protein structures, making the dough more extensible and reducing crumbliness of baked goods [58]. In gluten‐free systems enriched with animal or plant proteins, the combined use of proteases and TG can enhance structure: Proteases generate more reactive peptide chains, whereas TG cross‐links them into a stronger network, which increases dough resistance to mechanical stress and loaf volume [72]. Nevertheless, too intense proteolysis weakens the protein skeleton and may cause excessive spread and low volume.

TG is a protein cross‐linking enzyme that catalyzes acyl transfer reactions between glutamine and lysine residues, forming *ε*‐(*γ*‐glutamyl) lysine covalent bonds between protein chains [14]. In gluten‐free doughs enriched with milk, egg, or legume proteins, these cross‐links generate a more cohesive three‐dimensional protein network that partially mimics the viscoelastic behavior of gluten. Typical TG dosages of about 0.02%–0.05% (based on flour weight) have been reported to increase batter viscosity, improve gas‐cell stability during proofing and baking, and result in crumbs with higher porosity and lower hardness, as well as a slower increase in firmness during storage. Quantitative data from gluten‐free bread trials show that adding only 0.02%–0.05% microbial TG can increase loaf volume and reduce crumbliness by about 20%–30% compared with control breads without the enzyme [14, 68]. However, the effectiveness of TG strongly depends on the amount and type of available proteins: In low‐protein formulations or at excessively high enzyme levels, cross‐linking may produce overly rigid structures and reduce loaf volume, indicating that TG must be carefully optimized for each recipe.

Phytases that degrade phytic acid are widely used to increase mineral bioavailability in gluten‐free products. At the same time, phytase addition can have an indirect positive effect on texture by reducing protein–mineral crosslinking and improving water‐holding capacity of the dough, which favors higher loaf volume and softer crumb [68]. Other enzymes such as xylanases and cellulases, when used in small doses, break down hemicelluloses and cellulose, slightly liquefying the matrix and improving gas retention, but at excessive levels they may weaken the structure and promote collapse [38].

Overall, the best results are obtained when enzymes are used in combination and in synergy with sourdough. Modern studies show that combinations of amylolytic enzymes (*α*‐ and *β*‐amylase) with TG and gluten‐free sourdough can significantly increase dough viscoelasticity, specific loaf volume and crumb softness, while slowing down staling [14, 38, 66, 68]. For example, multienzymatic systems have been reported to increase loaf volume by roughly 10%–20%, reduce crumb hardness by 15%–30% and extend the fresh shelf life by 1–2 days compared with control breads without these additives [47]. Emerging process aids such as ultrasound applied during fermentation may further enhance enzyme action and moisture distribution, resulting in a more delicate crumb texture, although these technologies still require validation under industrial conditions [38].

The baking step represents the final and critical stage in determining gluten‐free bread texture. During baking, several transitions occur almost simultaneously: Starch granules gelatinize, proteins denature and aggregate, hydrocolloids hydrate and gel, and gas cells expand until the matrix sets [37, 43].

In gluten‐free formulations, where the structural network is mainly formed by starch gels together with added proteins and hydrocolloids instead of gluten, the time–temperature window in which gas expansion is balanced by matrix setting is much narrower than in wheat doughs. If the temperature rise is too rapid, the outer layers set prematurely and restrict expansion, resulting in low loaf volume and a compact crumb; if heating is too slow or the batter is too weak, gas cells may coalesce and collapse, producing large voids or a gummy core [43]. Several recent reviews emphasize that oven temperature profile, baking time, and the use of steam strongly influence crust formation, moisture gradients, and the final distribution of water between crust and crumb, and thus the subsequent evolution of hardness and staling [37, 73]. At the same time, many formulation studies on gluten‐free bread still apply “standard” baking programs without systematic optimization or even full reporting of baking conditions, which limits comparability between experiments and the translation of laboratory recipes to industrial scale [37, 71, 73]. Therefore, future research on gluten‐free bread texture should treat baking conditions as an equally important variable to be optimized, rather than a fixed background step.

### 3.4. Staling of Gluten‐Free Bread and Methods to Slow Down it

Staling of gluten‐free bread is a multifactorial phenomenon in which starch retrogradation, changes in the protein matrix, lipid crystallization, and moisture redistribution act simultaneously [74]. Because the crumb of gluten‐free bread is mainly supported by starch gels and added hydrocolloids rather than by a continuous gluten network, an increase in crumb firmness and a decrease in elasticity typically occur within the first 24–72 h of storage. As hardness increases and resilience and cohesiveness decrease, consumers perceive the bread as dry, crumbly, and stale [43, 44]. In other words, the faster loss of elastic recovery of the crumb is one of the key textural manifestations of staling in gluten‐free systems.

One of the main factors determining the rate of staling is the type and functionality of starches and flours used in gluten‐free formulations. Breads based on refined rice or corn starch often exhibit a rapid increase in crumb firmness due to the high tendency of their amylopectin to retrograde during storage [43]. By contrast, authors [19] demonstrated that partial replacement of corn or rice starch with sorghum flour slowed down crumb firming and improved sensory acceptability, which was attributed to the higher dietary fiber content and specific thermal properties of sorghum. Similar antistaling effects have been reported for gluten‐free breads fortified with fiber‐rich ingredients, which dilute the starch phase, bind additional water, and interfere with amylopectin recrystallisation.

Hydrocolloids are another key tool for controlling staling in gluten‐free bread. By increasing water‐binding capacity and batter viscosity, hydrocolloids assist to stabilize gas cells during baking and to retain moisture in the crumb during storage. Studies on corn‐ and rice‐based formulations have shown that supplementation with about 1%–2% HPMC (flour basis) can increase specific loaf volume and significantly slow down the increase in crumb hardness over several days of storage [43, 44]. However, these effects are strongly dose‐dependent: Excessive HPMC levels may lead to overly sticky or gummy crumbs and negatively affect mouthfeel, which underlines the need for careful optimization of hydrocolloid type and concentration for each flour blend.

Recently, additional strategies to slow down staling have been proposed, such as the use of lactic acid bacteria starters producing exopolysaccharides, biopolymer films, or active packaging systems with moisture‐regulating or antioxidant components [19, 43]. Although these approaches can contribute to improved moisture distribution and delayed firming, they also raise practical questions regarding process complexity, cost, and clean‐label positioning, so their application in commercial gluten‐free bread is still limited.

Overall, current evidence indicates that staling of gluten‐free bread results from complex interactions between starch composition, added fiber and hydrocolloids, protein level, and processing conditions. Future studies should therefore not only report crumb texture parameters (hardness, cohesiveness, and resilience) and moisture content over storage, but also use harmonized storage and testing protocols. This would allow a more quantitative comparison of antistaling strategies and facilitate the rational design of gluten‐free formulations with extended freshness.

### 3.5. Latest Technologies: Extrusion and Microencapsulation

In addition to the ingredient‐based approaches discussed above, several technological treatments of raw materials have been explored to improve the texture of gluten‐free bread. These methods are aimed at modifying flour functionality before dough mixing, thereby influencing dough rheology, gas‐cell development and the setting of the crumb during baking. In the last decade, the most intensively studied treatments in this context have been extrusion (pre‐extrusion of gluten‐free flours) and microencapsulation of functional ingredients.

Extrusion is a short‐term treatment of flour or starch at elevated temperature (typically 80°C–120°C) and pressure, followed by a sudden pressure drop at the extruder outlet. This process partially gelatinizes starch, denatures proteins, and breaks down large particles, which increases water absorption and can substantially alter pasting behavior [28]. When extruded flours (rice, corn, sorghum, and others) are incorporated into gluten‐free bread formulations, several studies report improved gas‐retention capacity of the dough and higher specific loaf volume, as well as a more open and uniform crumb structure [28, 75]. From a texture perspective, these effects are mainly related to the higher cold viscosity and earlier onset of gelatinization of the extruded starch fraction, which stabilizes gas cells during the critical stages of proofing and early baking. However, the impact is strongly formulation‐dependent, and the same treatment conditions do not necessarily yield the same textural benefits across different flour bases.

Extruded materials often exhibit increased cold viscosity and water‐binding capacity, which is generally favorable for specific volume and crumb softness [75]. At the same time, some authors point out potential sensory drawbacks: Excessively severe extrusion conditions may generate a characteristic “extrusion” flavor or aftertaste and darken the crumb [76, 77]. From a structural point of view, overprocessing can lead to the excessive destruction of starch granules and extensive protein denaturation, reducing the ability of the dough matrix to form a continuous network during baking and even decreasing loaf volume [78, 79]. This means that extrusion should be viewed as a fine‐tuning tool rather than a universal solution: Process parameters (temperature, moisture content, screw speed, and residence time) must be carefully optimized for each raw material to balance improvements in texture against possible sensory and structural drawbacks [80, 81].

The effect of extrusion is often enhanced when combined with other structuring agents. The simultaneous use of extruded flour and hydrocolloids (e.g., guar, xanthan gum, and alginate) has been shown to promote the formation of a particularly well‐developed and stable porous crumb structure [82]. In this case, hydrocolloids stabilize gas bubbles in a viscous, partially pregelatinized matrix, preventing coalescence and collapse. Nevertheless, excess levels of some hydrocolloids (for example, xanthan) can increase dough stickiness and negatively affect mouthfeel [83, 84], so optimization of both extrusion conditions and hydrocolloid dosage is required. Combinations of extruded flours with sourdough fermentation are also promising: Introducing starter cultures or yeast on extruded substrates has been associated with richer flavor and higher antioxidant content [85, 86]. Mechanistically, extrusion makes starch and proteins more accessible to enzymatic hydrolysis, whereas microorganisms produce additional bioactive compounds and exopolysaccharides that can further improve crumb softness and moisture distribution. These findings suggest that extrusion should be considered in an integrated way together with hydrocolloids, proteins and fermentation, rather than as an isolated process step.

Microencapsulation is another technology that can indirectly influence texture by allowing the incorporation of otherwise problematic functional ingredients in a way that minimizes their negative impact on dough structure and sensory properties. In baking, microencapsulation is typically applied to biologically active compounds such as vitamins, mineral supplements, fatty acids, probiotics and enzymes [81, 87]. The core idea is to protect these sensitive components from baking temperature, oxygen and moisture and to control their release. Studies have shown that microencapsulated additives can retain a much higher proportion of their activity during baking; for example, adding microcapsules containing fish oil and vitamin C preserved about 90%–95% of docosahexaenoic acid (DHA) after baking, whereas less than 50% remained when fish oil was added in free form [88]. At the same time, encapsulation prevented the typical deterioration in bread quality: Although free fish oil usually reduces specific loaf volume and worsens crumb structure, the microencapsulated fat combined with vitamin C kept volume and basic textural parameters close to control levels and effectively masked the fishy odor [89]. Thus, microencapsulation can act as a tool to maintain or even improve texture and sensory quality when incorporating bioactive ingredients that would otherwise destabilize the dough or impart undesirable flavors [89, 90].

In baking practice, the most common microencapsulation methods include spray drying, coacervation, extrusion‐based capsule formation, and ionic gelation [91, 92]. Each method has specific implications for capsule strength and behavior in dough. Capsules that are too fragile may break during mixing, releasing the core material prematurely, whereas excessively robust capsules may not release their contents during baking or digestion [93, 94]. Optimal systems are designed so that capsules open either at a specific stage of the technological process (e.g., during baking) or in a target region of the gastrointestinal tract (for probiotics). For instance, experimental incorporation of microcapsules containing *α*‐amylase has shown that the enzyme remains active for longer and starts acting on starch predominantly during baking, contributing to more uniform crumb loosening without compromising loaf volume [95].

Despite these advantages, microencapsulation has several limitations, including increased cost and process complexity. Additional equipment and encapsulating agents are required, which raises the price of the final product and may limit the applicability of this technology in low‐cost gluten‐free bread [73, 96]. On an industrial scale, dough mixing conditions must be adjusted to achieve a homogeneous distribution of capsules while avoiding their mechanical destruction; overly intense mixing may cause capsule breakage or localized agglomeration, leading to inhomogeneous texture. Furthermore, the proportion of microcapsules in the formulation must be kept within an optimal range, as excessive levels (e.g., > 5%–10% of dry matter) can reduce loaf volume and deteriorate crumb texture by introducing too many inert solid particles into the dough [96]. When appropriately designed and dosed, however, microencapsulation opens up new possibilities for enriching gluten‐free bread by vitamins, probiotics and enzymes without sacrificing texture, volume or consumer acceptance [73]. Future work in this area should combine technological optimization with cost–benefit analysis and sensory evaluation in order to identify economically viable strategies for routine use of microencapsulation in gluten‐free baking.

## 4. Innovations and Future Directions

The strategies discussed in this review demonstrate substantial progress in improving the texture of gluten‐free bread, but they differ markedly in terms of technological complexity, cost, and suitability for large‐scale production. The most established and industry‐ready options are still the use of hydrocolloids and protein fortifiers. Hydrocolloids are relatively easy to incorporate into existing formulations, are already widely available on the ingredient market and can often be dosed using standard dry‐mix systems. Numerous studies report 10%–30% increases in specific loaf volume and 20%–50% reductions in crumb hardness when appropriate hydrocolloids are used [17, 54]. At the same time, their effect is strongly dose‐ and system‐dependent: Suboptimal selection of type and concentration can lead to sticky or rubbery crumb, excessive moisture retention, or poor flavor release. From an industrial perspective, hydrocolloids are attractive because of their moderate cost and scalability, but they can conflict with “clean label” expectations if declared using unfamiliar E‐numbers. Recent work on plant‐based and cereal‐derived hydrocolloids and fibers therefore represents a promising direction for reconciling functional performance with consumer demand for simple, recognizable ingredient lists.

Protein ingredients provide a dual benefit: They contribute to structure formation and increase the nutritional value of gluten‐free bread. Animal proteins such as milk and egg proteins have consistently shown strong positive effects on loaf volume, crumb softness, and elasticity due to their foaming and film‐forming properties, sometimes yielding breads comparable with wheat counterparts in several texture attributes [22]. However, their use raises several practical issues. First, milk and egg are major allergens, and their inclusion restricts the target consumer group and requires clear allergen labelling. Second, animal protein isolates are relatively expensive and can significantly increase the production cost of gluten‐free bread [97]. Plant proteins (soy, pea, lupine, hemp, rapeseed, etc.) are attractive as vegan options and for improving amino acid balance, but they are often associated with beany or bitter off‐flavors and, at high levels, may stiffen the crumb because excess proteins compete with starch for water and hinder gelatinization [35]. This makes optimization of protein type and dosage critical and suggests that future research should focus on multiobjective formulation strategies that jointly maximize texture, nutritional value, flavor and cost‐effectiveness, rather than considering protein enrichment in isolation.

Enzymatic solutions and sourdough starters offer a more “biotechnological” route, as they modify the dough matrix in situ rather than merely adding structural fillers [98]. Sourdough‐based systems can naturally enhance flavor and aroma, contribute to a more elastic and cohesive crumb and prolong microbial shelf life due to acidification and the production of antifungal metabolites, but they also increase process complexity and time, requiring robust culture management and process standardization at industrial scale [99, 100]. Commercial enzyme preparations such as amylases, proteases, and TG can, at very low dosages, increase gas retention, improve crumb softness, and slow down staling without major changes in the ingredient list [69]. Their main limitations are the narrow optimal activity windows (pH, temperature, and water activity) and the risk of defects when overdosed (e.g., liquefied dough with excessive protease, and gummy crumb with too much amylase) [101, 102]. In addition, the regulatory status and labelling of enzymes differ between jurisdictions: In many markets, they are treated as processing aids and need not be declared on the label, whereas in others, certain enzymes or production strains require explicit authorization. At the same time, consumers who are sensitive to “technological” interventions sometimes perceive enzymes as less natural, which may clash with clean‐label positioning. Nevertheless, multienzymatic systems combining amylolytic enzymes with TG and gluten‐free sourdough have emerged as one of the most effective approaches, achieving simultaneous increases in loaf volume (≈10%–20%), reductions in crumb hardness (≈15%–30%) and extensions of fresh shelf life by 1–2 days compared with control breads [70, 71, 103]. These results highlight the need for closer collaboration between enzyme suppliers and bakeries to translate laboratory protocols into robust industrial processes.

Raw‐material extrusion and microencapsulation represent a newer generation of technologies that modify ingredient functionality prior to dough mixing. Pre‐extrusion of gluten‐free flours can fundamentally alter starch and protein properties, increasing cold viscosity, water‐binding capacity, and gas‐holding ability, which in many studies leads to higher specific loaf volume and a more open, uniform crumb [28, 75]. Microencapsulation, in turn, allows the incorporation of otherwise problematic components (e.g., omega‐3 fatty acids, vitamins, probiotics, and enzymes) while protecting them from thermal degradation and limiting their negative effects on dough structure or flavor [81, 87]. This makes it possible to design “two‐in‐one” products that are both texturally acceptable and enriched with bioactive components. However, both technologies face significant barriers to widespread industrial adoption. Extrusion requires capital investment in specialized equipment and entails high energy costs; its impact on flavor can be negative under harsh conditions, and it may be difficult for small and medium‐sized bakeries to integrate an extrusion line into existing process flows. Microencapsulation adds extra processing steps and encapsulating materials, increasing ingredient and operational costs; it also raises regulatory questions related to the approval and labelling of encapsulating agents and the substantiation of health claims. For both technologies, laboratory‐scale successes are not always directly transferable to industrial scale, and additional work is needed on process design, cost–benefit analysis, and consumer acceptance of “fortified” gluten‐free breads [77, 88].

When comparing the overall contribution of different strategies, hydrocolloids and enzymes primarily affect crumb softness and freshness (i.e., they are powerful tools to manage staling), whereas proteins and extrusion mainly influence specific loaf volume and crumb porosity by creating or reinforcing the structural framework. Microcapsules mainly contribute to quality stability and nutritional value by preserving sensitive components and masking undesirable flavors. In practice, the most promising gluten‐free breads combine several of these approaches—for example, a formulation including HPMC, egg white, TG, and sourdough can match many sensory attributes of wheat bread [73]. Yet such multicomponent systems are complex and costly, so real‐world manufacturers must balance performance against ingredient cost, process complexity, and target price point. For mass‐market products, relatively simple combinations (e.g., one or two hydrocolloids plus a single enzyme and moderate protein enrichment) may offer the best compromise between technological performance and economic feasibility.

From an innovation perspective, several emerging directions deserve attention. One is the development of clean‐label gluten‐free breads that rely on minimally processed flours, natural fibers, and in situ fermentation‐derived exopolysaccharides instead of synthetic or highly modified additives, in line with current consumer trends [70, 72]. Another is the use of advanced processing and design tools—such as 3D/4D printing of dough systems, digital twins for baking processes, and data‐driven optimization of formulations—to tailor crumb structure and texture more precisely [30, 31]. At the same time, consumer studies indicate that many individuals following a gluten‐free diet still perceive gluten‐free bread as dry, crumbly, and expensive, and are cautious about unfamiliar ingredients or technologies [11, 67].

The use of lesser‐known regional specific crops, such as teff in the gluten‐free bread technologies, allows for the creation of a wide range of bakery products.

*Eragrostis tef (Zucc.) Trotter*, commonly known as teff, is an ancient, nutrient‐rich, and gluten‐free cereal native to Ethiopia and Eritrea [104]. Native teff starch, characterized by small granule size and high amylopectin content, is suitable for several industrial uses; however, it often requires modification to enhance its thermal stability, solubility, and resistance to digestion [105]. Multiple studies report that teff is naturally gluten‐free and provides slowly digestible carbohydrates (often with a low glycemic index), dietary fiber, essential amino acids, vitamins, minerals, and diverse polyphenols [106]. Teff′s nutritional value is superior compared with that of other gluten‐free grains, like quinoa and rice, with teff being richer in fiber, iron, and calcium than flours and other gluten‐free products [107]–109].

Therefore, future research should not only focus on improving instrumental texture parameters but also systematically address industrial scalability, production low cost, local raw materials accessibility, regulatory compliance (especially for enzymes, novel encapsulating materials, and health claims), and consumer acceptance. Only by integrating ingredient functionality, process engineering, economics, and consumer science will it be possible to move gluten‐free bread from a “compromise product” towards a mature category that reliably meets both nutritional and sensory expectations in real market conditions.

## 5. Conclusions

The lack of gluten in dough inevitably leads to typical quality defects of gluten‐free bread—low specific volume, dense and crumbly crumb, and rapid staling that is related to no continuous viscoelastic protein network to stabilize gas cells and retain moisture. Over the past decade, it has become clear that these shortcomings cannot be eliminated by a single component or processing technology. Instead, gluten‐free bread quality depends on the valuable action of specific ingredients: polysaccharides, proteins, hydrocolloids, emulsifiers, enzymes, and others, as well along with the intervention approaches: fermentation, microencapsulation, and process conditions.

Natural polysaccharides, particularly plant‐derived hydrocolloids, provide both technological and nutritional benefits by improving water binding, viscosity, and stabilizing gas bubbles. Microbial hydrocolloids derived from fermentation are effective even at low dosages and can act synergistically with cereal or plant fibers to stabilize the crumb. Seaweed‐based hydrocolloids have strong gel‐forming properties that make them promising for specific applications, for example, in flatbreads or products with high moisture. Protein‐based or animal‐derived hydrocolloids such as gelatin and milk proteins can form thermally stable gels and protein–polysaccharide complexes that support the crumb structure.

Starters and enzymes are key biotechnological tools for modifying gluten‐free dough rheology and sensory quality. The most commonly used enzyme groups are amylolytic, proteolytic, TG, phytase, and enzymes produced during sourdough fermentation. Combinations of amylolytic enzymes (*α*‐ and *β*‐amylase) with TG and gluten‐free sourdough can increase dough viscoelasticity, specific loaf volume, and crumb softness while slowing down staling.

Meanwhile, oven temperature profile, baking time, and the use of steam strongly influence crust formation, moisture gradients, and the final distribution of water between crust and crumb, and thus the subsequent evolution of hardness and staling.

The crumb texture parameters (hardness, cohesiveness, and resilience) and moisture content over storage, also use harmonized storage and testing protocols are important approaches for staling of gluten‐free bread. This would allow a more quantitative comparison of antistaling strategies and facilitate the rational design of gluten‐free formulations with extended freshness.

The extrusion (pre‐extrusion of gluten‐free flours) and microencapsulation of functional ingredients approaches allow to improve the texture of gluten‐free bread. The simultaneous use of extruded flour and hydrocolloids forms a particularly well‐developed and stable porous crumb structure. Additionally, combinations of extruded flours with sourdough fermentation, specifically introducing starter cultures or yeast on extruded substrates provide richer flavor and higher antioxidant content. Microencapsulation permits enriching a gluten‐free bread by vitamins, probiotics, and enzymes without sacrificing texture, volume, or consumer acceptance. Combination of raw‐material extrusion and microencapsulation can design “two‐in‐one” products that are both texturally acceptable and enriched with bioactive components.

Future research on gluten‐free bread texture should move beyond simply screening new additives and focus on integrated, multiobjective optimization. This includes rational selection and combination of flours, hydrocolloids, proteins, and enzymes for specific product concepts; systematic optimization of fermentation and baking conditions; and simultaneous consideration of sensory quality, nutritional value, cost, regulatory constraints, and consumer perception. Particular attention should be paid to clean‐label approaches (e.g., use of fermentation‐derived exopolysaccharides and minimally processed fibers) and to scalable process innovations that can be realistically adopted by industry. By combining advances in food chemistry, biotechnology, process engineering, and consumer science, the next generation of gluten‐free breads can be designed to be not only technically sound and nutritionally adequate, but also economically viable and widely accepted by the potential gluten‐free diet consumers.

## Author Contributions

**Zaripa Alibekova** and **Ravshanbek Alibekov:** conceptualized and written the first draft of manuscript; **Meruert Bayisbayeva**: analyzed the data and interpreted findings; **Rosnah Shamsudin**: edited and provided the resources; **Asem Bakhtybekova**: visualized and took charge of the software components; **Zhambul Aimenov:** supervised the overall work progress.

## Funding

No funding was received for this manuscript.

## Disclosure

All authors have read and agreed to the published version of the manuscript. The authors have reviewed and edited the output and take full responsibility for the content of this publication.

## Ethics Statement

The authors have nothing to report.

## Consent

The authors have nothing to report.

## Conflicts of Interest

The authors declare no conflicts of interest.

## Data Availability

The datasets supporting the statements and conclusions of this review will be made available by the authors upon request.
